# Health‐care professionals’ experiences of patient participation among older patients in intermediate care—At the intersection between profession, market and bureaucracy

**DOI:** 10.1111/hex.12896

**Published:** 2019-05-24

**Authors:** Linda Aimée Hartford Kvæl, Jonas Debesay, Asta Bye, Astrid Bergland

**Affiliations:** ^1^ Department of Physiotherapy, Faculty of Health Sciences Oslo Metropolitan University Oslo Norway; ^2^ Department of Nursing and Health Promotion, Faculty of Health Sciences Oslo Metropolitan University Oslo Norway; ^3^ European Palliative Care Research Centre (PRC) Department of Oncology Oslo University Hospital and Institute of Clinical Medicine University of Oslo Oslo Norway

**Keywords:** empowerment, geriatrics, patient participation, professional work, quality of health care, rehabilitation

## Abstract

**Background:**

Patient participation is a key concern in health care. Nevertheless, older patients often do not feel involved in their rehabilitation process. Research states that when organizational conditions exert pressure on the work situation, care as a mere technical activity seems to be prioritized by the health‐care staff, at the expense of patient involvement.

**Objective:**

The aim of this article is to explore how health‐care professionals experience patient participation in IC services, and explain how they perform their clinical work balancing between the patient's needs, available resources and regulatory constraints.

**Design:**

Using a framework of professional work and institutional logics, underpinned by critical realism, we conducted semi‐structured interviews with 18 health‐care professionals from three IC institutions.

**Results:**

IC appears as an important service in the patient pathway for older people with a great potential for patient participation. However, health care staff may experience constraints that prohibit them from using professional discretion, which is perceived as a threat to patient participation. Further, they may adopt routines that simplify their interactions with patients. Our results call for more emphasis on an individualized rehabilitation process and a recognition that psychological and social aspects are critical for patient participation in IC.

**Conclusion:**

Patients interact in the face of conflicting institutional priorities or protocols. The study adds important knowledge about the practice of patient participation in IC from a front‐line provider perspective. Underlying mechanisms are identified to understand and recommend how to facilitate patient participation at different levels in narrowing the gap between policy and clinical work in IC.

## 1 INTRODUCTION

Over recent decades, political changes have strengthened the patient's position through laws and regulations that require the implementation of patient participation in health care.^1^, ^2^, ^3^ Patient participation implies that the patient's capabilities, needs and goals should individualize the health care interventions.^3^, ^4^, ^5^, ^6^ This may be a concern in the care of frail older patients with multiple and chronic diseases, a group that is increasing globally.^7^ As the length of stay in hospitals in the Western world is significantly reduced,^8^, ^9^, ^10^ the establishment of intermediate care (IC) has been one of the initiatives to bridge the treatment gap between hospital and home for older frail patients. The IC services provide rehabilitation for a limited time period,^11^, ^12^ often in specialized units in the municipality.

A concept analysis defines patient participation in IC as “a dynamic process emphasizing the person as a whole, focusing on the establishment of multiple alliances that facilitate individualized information and knowledge exchange, and ensuring a reciprocal engagement in activities within flexible and interactive organizational structures.” (p1343)^13^ IC services are meant to be holistic, taking into consideration psychological, social and physical aspects of function.^14^ Yet, studies indicate that frail patients often do not feel involved in rehabilitation.^15^, ^16^ Identified challenges are organizational and professional collaboration, but there exists little information about how these challenges impact clinical work.^17^, ^18^, ^19^

In Norway, the health care system is predominantly state‐funded and divided into specialist and primary health care. Primary care includes home‐based services and nursing homes, while the specialist health care involves the state‐owned hospitals organized in four regional health authorities.^20^ Rehabilitation for older people is delivered at both primary (eg, home‐based reablement/physical therapy) and specialist (eg, specialized hospital units after stroke) levels. In addition, similar to other countries, Norway has the last two decades developed nurse‐led intermediate rehabilitation based on shared care between specialist and primary care.^21^

In many Western countries, including Norway, New Public Management‐inspired (NPM) reforms of health and social care gained strong political impact throughout the 1990s.^22^ The main idea is allowing the government to retain ownership of the company but still enable it to be run as a private sector company.^23^ The emphasis on cost‐effective services along with the policy of deinstitutionalization has increased the turnover of hospital patients and the work‐load in the municipalities,^24^ and reduced the number of nursing home places and similar housing facilities. Furthermore, decentralization has increased the municipalities responsibility to develop coordinated patient pathways across care levels.^25^ In line with the ideas of NPM, a purchaser‐provider model has been developed in the districts of Oslo to ensure a distinction between those who assess the need for services and those who provide the services.^26^, ^27^ This implies that the purchasers (the municipal districts) have the administrative authority to assess and approve the services, while the providers (staff in IC) have minimal influence on the administrative decisions other than performing the services.

### 1.1 Professional work and conflicting institutional logics

Eliot Freidson^28^ divides the working constraints in professional work into three distinct ideal institutional logics based on different assumptions: market, bureaucracy and professionalism. While the market logic celebrates competition and cost reduction, the bureaucratic logic invokes the virtue of efficiency through standardized procedures or routines. Freidson introduces professionalism as the third logic that promotes health‐care professionals’ commitment to quality in work. An underlying assumption within professionalism is that a profession has monopoly of expertise based on esoteric knowledge. Closely related to having monopoly of expertise is the professional discretion. While professional discretion is necessary in tailoring rehabilitation, it may challenge the notion that efficiency is predominantly gained by standardization.^28^

In human service organizations, health‐care professionals have room for interpretation to make strategic choices in terms of how to implement IC policies and guidelines. In light of this, the institution is an arena where different values and norms compete.^29^ Within these structures, health‐care professionals face work conditions that require adaptations and improvisations using discretion in their meetings with patients. Michel Lipsky provides relevant aspects for understanding the professionals’ experiences and attitude. He describes how front‐line providers face conflicting priorities because their tasks might be characterized by tension between the patients’ needs, available resources and regulatory constraints, that is conflicting institutional logics. The health‐care professionals who meet the patients face to face are those who carry out policies decided at a higher level, trying to find acceptable strategies for implementation in practice.^30^ The three ideal institutional logics can provide a useful lens for understanding the underlying structures of practical work for health‐care professionals in IC. However, although these logics conceptually tend to be held separate, which creates a fragmented understanding of health care,^31^ it is important to emphasize that in practice they are entangled.^32^ In addition, the choice of interviewing health‐care professionals means that the professional perspective is a point of departure in our study.

### 1.2 Aim of the article

Successful patient participation within IC services is associated with satisfaction with health‐care services,^33^, ^34^, ^35^ a lower number of readmissions,^16^, ^36^, ^37^ better treatment outcome^24^, ^25^, ^38^ and has the potential to prevent the need for nursing home placement.^39^ Further, patient participation is shaped by organizational structures,^40^ the patients’ condition,^41^ resources,^11^ staff attitudes^42^, ^43^ and support from relatives.^44^, ^45^ Still, there is great variation in how patient participation is managed and experienced by patients and relatives.^15^, ^16^, ^46^, ^47^, ^48^ Thus, health‐care professionals in IC need to have extensive skills within geriatric rehabilitation including patient participation and communication techniques, taking into account the complex needs of older patients whose health status often fluctuates.^15^, ^16^, ^49^

The aim of this study is to explore how health‐care professionals in IC services experience patient participation, and explain how they perform their clinical work balancing between the patient's needs, available resources and regulatory constraints. The study adds important knowledge about how to facilitate patient participation within conflicting institutional logics. By exploring underlying mechanisms through the lens of professional work, we hope to provide useful information in decreasing the gap between policy and clinical work in IC.

## 2 METHOD

The study employs a qualitative approach with a critical realism‐inspired philosophical framework. Social phenomena exist in an open system where mechanisms interact at different “layers of reality.” While structure is the recurrent patterned arrangements, which increase or decrease the opportunities and choices available, the agency implies the individual capacity to act independently and to make own free choices.(p448)^50^ The critical realist intends to reveal the underlying structures and mechanisms in order to explain social events and suggest recommendations to address social problems.^51^, ^52^, ^53^

### 2.1 Settings

The city of Oslo, Norway, has synchronized all short‐term rehabilitation services into four major institutions in order to meet demographic changes. The IC institutions are organized and managed by the Nursing Home Agency in Oslo. The districts purchase IC services in accordance with the patients’ needs; thus, the patients only pay a small deductible fee. The study was conducted in three out of the four institutions representing 75% of the districts in Oslo. Patients in IC typically receive medical treatment, social care and physical training in order to manage activities of daily living, a home visit with an occupational therapist, and follow‐up services from the district after discharge to home. It is the municipal district coordinator that assesses and approves an IC‐stay in collaboration with patient and relatives. The initial family meeting is supposed to be held within the first 2‐3 days of the patient's IC‐stay, in order to make a follow‐up plan. The municipal district coordinator and the patient's relatives are invited, in addition to the patient and the interdisciplinary IC team. The patients in IC often have complex health issues.

### 2.2 Participants

We included a strategic sample of 18 health‐care professionals, six from each IC institution, 15 women and three men, all permanent employees for a minimum of one year. Their ages ranged from 28 to 63 years. Average age was 43 years. The participants represent the interdisciplinary team in IC: nurses (3), doctors (3), occupational therapists (3), nursing assistants (3), physical therapists (3) and municipal district coordinators (3). Most had extensive work experience with an average of 14 years. Seven of the participants were immigrants from Somalia (2), Italy (1), Pakistan (1), Russia (1), Finland (1) and Germany (1).

### 2.3 Data collection

Health‐care professionals suitable for inclusion were informed and asked to participate by the first author. The participants represented six professions from each IC institution. However, they were not necessarily working together as a team, rather representing different units within the institution. The participants were chosen due to diversity in age, nationality and clinical experience. We conducted individual interviews (September 2017‐February 2018) using a semi‐structured interview guide (see Table 1), supported by follow‐up questions to initiate thick descriptions regarding how the participants experience patient participation in IC.^54^ The interviews were face‐to‐face and took place in the participant's workplace. Most of the interviews lasted one hour and were performed by the first author who has a background as a physical therapist and broad clinical experience within geriatric rehabilitation. All interviews were audiotaped and transcribed verbatim by a professional transcriptor.

**Table 1:** Interview guide

The interview takes place at the IC institution, in a room where it can be spoken undisturbed, and where the conversation can be recorded. The participant is initially informed about the project and then gives her/his written consent. The interview will be relatively open, but the following five thematic areas will be highlighted. The sub questions serve as a check list for the interviewer to ask, if the participant does not touch these areas throughout the conversation. Participation of patients in the clinical everyday life of IC Can you please describe how the department cooperates with the hospital to facilitate patient participation during transition from hospital to IC? Please give an ex. Can you please describe how the patient and relatives are involved in the family meeting? Can you please describe how you experience patient participation during the IC process? Eg meals, physical training, morning care, shared decision making. Please explain Can you please describe how the department cooperates with the municipal districts to facilitate patient participation during transition from IC to home? Please give an ex. The role/contribution of relatives in IC Can you please describe in what way relatives influence the content in the IC service? Please give an ex. Can you please give examples of what relatives do to promote patient participation? Can you please describe in what way relatives should be involved? Please give an ex. Understanding and use of the term patient participation Can you please describe how you understand the concept of patient participation? Please give an ex. Can you please describe the impact of patient participation in geriatric patients and their relatives' experience of their situation? In your opinion, how is patient participation related to quality of care? The participation of older patients in IC Can you please describe in what way patients may be able to influence the patient pathway? º Patients' opportunity for participation in treatment? º Patients' opportunity for participation in decision making? º Patients' opportunity for participation in the transitions? Can you please describe in what way (and degree) you think they would like to be involved? Can you please describe how do you think older people can participate? Do you think they should have more influence than they have today? How? The impact of framework and organization Can you please describe your opinion about the role of framework and organization (eg ICs' design, location and facilities, budget, number of employees, working hours, meeting structure and work routines) for patient participation in practice? Can you please describe the factors of importance for the geriatric patients' voice to be heard? Can you please describe the factors that might inhibit patient participation in practice? Please describe how you experience patient participation is anchored at the management level? Please describe how you experience patient participation is anchored across service levels? Open questions are asked first, but the researcher can also ask about the descriptions given by others and which the participants can comment on. At the end, sum up and check if the participants are correctly understood. Open up for additional information: "Is there anything I haven't asked about that you think may be of importance?"

### 2.4 Data analysis

The interviews were structured using the software HyperRESEARCH and analysed using thematic analysis based on Braun and Clarke.^55^ Thematic analysis is widely used and compatible with a realist approach. First, the authors read the interviews in an active way, searching for “demi‐regularities” or frequently reproduced patterns across the data material. Then, the material was coded by the first author, extracted from quotes about patient participation and organized into meaningful groups. Critical realism‐informed categories like “structure” and “agency” served as a starting point for defining the initial themes (Table 2). The software HyperRESEARCH was also used to identify the most dominant codes. After identifying the initial themes, the process of abduction allowed us to use the theoretical framework as a lens in abstracting the data into the further analysis, resulting in three main themes (Table 3). Defining, reviewing and naming themes led to discussion between the authors, with backgrounds in physiotherapy (2), nursing (1) and nutrition (1), all researchers with long clinical and/or extensive research experience in geriatric health care. In order to preserve variability, reflexivity and to establish credibility, all authors carried out the analysis.^56^ To enhance validity, the first author discussed the results with staff and leaders from one IC institution and one municipal district, as well as with personnel working on professional development within institutions and home‐based services in the municipality.

**Table 2:** Example of coding procedure

Quotes about patient participation	Code	Group	Initial theme
“The patient discharge from hospital is a fast process. The assessment is often incomplete, it lacks information, characterized by urgency. The economy is everything. When the hospital reports the patient ready for discharge, we (the districts) have to offer a place, or pay daily expenses for patient overlays. They send people out in full delirium, and services like IC are left with complex patient cases” (district coordinator, 37 years)	The cooperation with hospitals	Structure	Standardization at the cost of individualization
“I'm not supposed to fix the patient's problem, but the patient should be allowed to be himself in this process, be allowed to be human, not just a patient. The person should not be a product, but should be allowed to indicate what is important for him/her, and then it is my duty to give support beyond what is being said. And not only ‘I hear what you say, but it's the wrong time and place to have those feelings, now we will focus on your hip fracture and managing the toilet visits’, eg” (occupational therapist, 30 years)	To see the person behind the diagnosis	Agency	Patient participation as empowerment in rehabilitation

**Table 3:** Results of the analysis

Initial themes	Main themes
1. At the premises of the municipal districts	The purchaser‐provider model and standardization of patient participation
2. A predetermined pathway in a bureaucratic maze	
3. The initial family meeting as a crossroad	
4. Standardization at the cost of individualization	
5. Patient participation as disclaimer	
6. A place to be stored instead of rehabilitated	IC as a storage facility losing its rehabilitative/preventive function
7. Lowest effective instead of best effective care level	
8. Relatives as a challenge and a resource	
9. Discharged to home before they are ready	
10. The constant lack of time and resources	
11. Prioritizing technical over relational tasks	The lack of professional discretion and empowerment of health‐care professionals
12. Reorganization without front‐line providers’ involvement	
13. Patient participation as empowerment in rehabilitation	
14. “I can do more than make sandwiches”	
15. The lack of professional discretion in everyday practice	

## 3 RESULTS

The analysis resulted in three themes: “The purchaser‐provider model and standardization of patient participation,” “IC as a storage facility losing its rehabilitative/preventive function” and “The lack of professional discretion and empowerment of health‐care professionals.”

### 3.1 The purchaser‐provider model and standardization of patient participation

A good collaboration between the district and IC is crucial to facilitate patient participation. The participants reported that one of the main concerns in order to preserve patient participation in IC was the process of working together with the differently organized municipal districts. The participants described different cooperation models with the districts. The proactive municipal districts actively engage in the patient pathways through face‐to‐face meetings with the patients and their relatives, working in teams with the staff in IC, valuing their observations and opinions. In the opposite case, the districts were described as non‐existing, just providing information about what happens next for the patients, leaving the health‐care professionals in IC with restricted professional discretion. One nurse (31 years) stated:

The districts are the customer, the purchaser, they've ordered a service that we have to deliver. But the collaboration with the districts vary a lot. Some places it is non‐existent. Thus, it becomes difficult to provide a good service or to tell the patient what the plan is. So, it's absolutely wonderful when you have good collaboration, with initial family meetings to clarify expectations, and consensus regarding goals and tasks.

Thus, clear lines in the collaboration between IC and the districts were called for.

Staff expressed that patient participation in IC is a holistic process taking into consideration both physical, social and psychological aspects. Thus, establishment of good alliances with patients and relatives is considered necessary in order to obtain trust and team‐work. However, the participants highlighted that the overall bureaucratic system does not facilitate patient participation. As a patient, you have to fit the system and within the boundaries of normality to be perceived as deserving. Often this is all about physical criteria, at the cost of psychological and social considerations. One of the district coordinators (34 years) stated:

I understand the importance of the patient's voice being heard. No one fits into a box, I understand the thinking. But I see that real patient participation is difficult to achieve in a health‐care system that is built up like ours. These are the services we have! The day center has a specific structure that you have to fit within. And within IC there are other criteria. As a patient, you might say what you would like, but whether you ever receive it, is not up to you.

Thus, patients might be discharged to home too early, due to high pressure from hospitals.

Staff reported that the family meetings are highly appreciated with potential to promote patient participation. They said that family meetings should be characterized by respect and empathy, information and knowledge exchange, and real, alternative potential outcomes. It was necessary that the family meeting was held early in the process to clarify expectations and make a rehabilitation plan. However, of greater importance was the participation of relevant members, the patient, the relatives, the IC team and the district coordinator. Only the district coordinators can give realistic information regarding follow‐up services, and the staff in IC are not allowed to give any promises regarding these issues. In situations with some relevant members missing, the health‐care professionals experienced the family meeting as an organizational duty and not as a forum for genuine patient participation.

If the initial family meeting was to be of real value, the municipal district coordinator should be present. It is an opportunity to involve relatives, but you may not know anything concrete about follow‐up services and the patient pathway. And that's what the relatives really want to find out about (physical therapist, 32 years).

### 3.2 IC as a storage facility losing its rehabilitative/preventive function

Rehabilitation implies assisting the patients' own efforts in achieving the optimal level of coping and functional ability, independence and social participation and co‐production.^57^ In order to do so, the health‐care professionals stated that patients benefit from being in an atmosphere with the right professional mindset and fellow patients in similar situation in order to get motivated and feel relatedness. They felt that too many patients in the ward waiting for nursing home may inhibit patient participation.

People are waiting in line for a stay in a nursing home. And the politicians, I don't know where they take it from, but two years ago they decreased the number of places in long‐term care. But we, the health‐care professionals working on the floor, know people are waiting (nursing assistant, 52 years).

Thus, IC loses its rehabilitative function as it is also used as a place to house people waiting for long‐term care and/or construction of housing.

Another issue raised by the participants is the fact that being in IC is associated with a passive existence. Due to a feeling of constant lack of time and resources, there is nothing going on for the patients, except standardized routines like meals and occasional training. One of the physical therapists (46 years) said:

It’s an artificial situation. We rehabilitate in a place where you get so much service. One thing is that they have nothing practical to do. They just sit in their chairs, they do nothing but brush their teeth, go to the toilet, morning care… They do not contribute during meals. They get all the food served. So, there are no activities here that they participate in. Thus, they rapidly become passive, here too, at a rehabilitation post.

The professionals wanted more time to talk and engage *with* the patients in daily activities, rather than doing things *for* the patient. At the same time, some of the participants argued that IC is not a hotel, they are not there to be fixed, and the patients must also take responsibility for their own rehabilitation.

### 3.3 The lack of professional discretion and empowerment of health‐care professionals

Most of the health‐care professionals were concerned about the lack of time and resources for engaging with the patients in IC. The participants described that they spend a lot of time documenting, reporting and filling out required forms. One of the doctors (51 years) said:

But in order to prioritize, what comes last? It is to see the patient, it depends on time, and time is always lacking. Interpersonal, to meet someone who has arrived, who has been transferred maybe seven times, is not particularly satisfactory. I should have more time, I use 85% of my day staring into a computer, documenting.

As a strategy, care as a mere technical activity or routine seems to be prioritized when organizational conditions exert pressure on the work situation. Several participants felt this as an organizational devaluing of their work. One of the nursing assistants expressed this:

Within our technical and routinized workday, we forget about the patient. But there is so little focus on the mental and social values in our education. It's all about physical criteria. We see the disease but not the person behind it. I believe that if we increase our holistic understanding, it will also become more natural for us to let the patients decide (nursing assistant, 45 years).

We thus suggest more emphasis on the recognition that psychological and social aspects are critical for patient participation in IC.

The participants experienced constraints that prohibit them from using discretion, perceived as a threat to patient participation. They stated that their professional observations of the patients’ need in IC need to be taken more seriously.

The districts are the ones who will provide follow‐up services, so we are not allowed to recommend anything, and I think that's weird. Because we are the ones who observe and assess the patients every day. Yes, that's what we feel, that our observations have no value, in a way. The things we say do not matter… (nurse, 60 years).

The restricted professional discretion leaves little room for individualized rehabilitation. In practice, the participants must balance their work between regulatory constraints, limited resources and the patients’ needs.

## 4 DISCUSSION

Our findings indicate that there exists a complex relationship between professional work and the logics of market and bureaucracy. Initially, we will discuss the cooperation with the districts and the impact of routines and standardization in the light of NPM‐corporatization. Then, we will show how the market‐inspired policy of deinstitutionalization may explain “a missing link” in health‐care service delivery expressed as the IC being a place to store patients. Finally, the importance of professional discretion will be explored in order to promote quality in work and patient participation.

The NPM has two basic pillars, one in the market logic with an emphasis on competition and production, the other in the bureaucratic logic celebrating efficiency and standardization,^58^ challenging the professionalism as the third logic.^28^ The health‐care professionals highlighted that the cooperation with the districts varied extensively and that the quality of this collaboration had a great impact on the patient participation. Traditionally, the local authorities in Norway have been autonomous, though monitored by the central government, in organizing the services in line with local conditions.^59^ This has given a range of different service profiles in geriatric care.^60^ In addition, as a result of the NPM‐reforms and the corporatization, the diversity in service delivery is even more complex, ranging from public administrations and enterprises via mixed public/private organizations to private business institutions. Consequently, the districts have considerable institutional choice among different models of public service provision and must take strategic decisions on the best mix.^23^ To adopt the different practices, a collaboration model between IC and the districts might be implemented to create predictability and clear lines of responsibility in order to ensure participation in the patient pathway. To give an example, as a standard routine, the district coordinator could be required to participate in the initial family meeting in order to, at least, give information about follow‐up services. This is a way of involving the patient at a minimum low level, without any additional economic costs, and should be possible even within tight economic and structural frames.

The purchaser‐provider model ensures a distinction between those who assess the need for services and those who provide the services. By outsourcing the services, the idea is to secure the patients’ rights by controlling providers, which requires quasi‐markets and budgets.^59^ In the purchaser‐provider model, IC‐staff have limited influence on the administrative decisions other than performing the services ordered by the district. They are nevertheless held responsible for meeting the patient's needs and for documenting their work progressively. Thus, in situations with limited professional discretion, bureaucratic standardization might be prioritized over individualized rehabilitation.^24^

According to Lipsky, standardization of health‐care services can be seen as a strategy in order to survive balancing work between regulatory constraints, limited resources and patients’ needs.^30^ In addition, as a patient in the standardized pathway, you have to fit the system based on predominantly physical criteria, often at the expense of mental and social aspects. In a free market, the customer has the power to choose and complain about the service. In the quasi‐market system, the patients have not the same ability to choose, and the outsourcing of services can make flexibility more difficult. This is also in line with the bureaucratic logic of Freidson, which invokes the virtue of efficiency through standardized procedures.^28^ Thus, professionals may experience constraints in exercising discretion and thereby limiting patient participation. A recent evaluation of three decades with NPM in UK, states that this way of controlling management leads to an increased bureaucratic and expensive administration, at the cost of overall service resources.^61^

Most participants believed that the politicians’ policy of deinstitutionalization does not meet the overall needs of older people as a heterogeneous group. Politicians have reduced the number of places in nursing homes and extended and specialized the home care services. Internationally, having people remain in their own homes for as long as possible, is favoured by policymakers, health providers and by many older people,^62^ and is in line with the aim of IC, that is to help people back home again. However, among the oldest old, there might be a possibility that they no longer are able to be at home and feel safe anymore. Then, it should be possible to be granted a place in a nursing home, without fighting to be qualified, or wait for a long time in an IC facility. A cost‐saving policy is influenced by what Freidson (2001) describes as the market logic, which celebrates competition and cost reduction.^28^ However, in order for this market logic to function there must be real competition.^63^ Our findings indicate a situation characterized by a lack of nursing home places. As a result, IC loses its rehabilitative function as it is largely used as a place to house people waiting for long‐term care. A recent public survey (2018) reveals that 44% of the municipalities in Norway have patients waiting for nursing home, or a similar housing, and out of these almost 70% are blocking a bed in IC.^64^ The lack of nursing homes inhibits IC to fulfil its potential regarding patient participation.

Being in IC is associated with a passive existence. Due to a feeling of a constant lack of time and resources, there was little going on for the patients, except standardized routines. Patient participation takes more time,^47^ “doing for” requires less time than “doing with.” This is in line with previous IC‐research, which states that when organizational conditions exert pressure on the work situation, care as a practical activity seems to be prioritized,^11^ at the cost of patient involvement.^65^ At the same time, some of the participants argued that IC is not a hotel, they are not there to be fixed, and the patients must also take responsibility for their own rehabilitation. According to Mik‐Meyer (2017), the market context, in addition to a lowering of costs, is also about strengthening patient agency, to ensure the patients’ rights. She argues that soft power regulates the quality of patient participation in practice.^66^ The NPM and public governance can be considered as a modified version of the market. In this respect, the mechanisms of the market logic still apply to a certain extent, although the patient is not a paying costumer in the Norwegian welfare system. The patients are expected to take on an active role, voice their needs and make choices in the rehabilitation process.^2^ Then again, the health‐care professionals in IC have to transfer their expertise to the patients, educate them into experts. Obviously, not all older frail patients are able to take on this demanding role, leaving little room for the vulnerable patient unable to formulate a solution to the problem. Respectively, as the results indicate, it can be quite demanding for the health‐care professionals as well, who then have to engage with the patients in a more coaching way.^66^ The ethos of patient participation is also rooted in the knowledge, attitude and a recognition that psychological and social aspects are critical for patient participation, which should be implemented as part of the organizational training.

The participants described that they spend a lot of time documenting and filling out required forms at the cost of time spent with the patients. Freidson (2001) promotes the professional commitment to quality in work in his third logic: professionalism. As the market logic seeks efficiency and the lowest possible costs, it may be tempting to choose cheap solutions, with a decline in quality as a result. Within the market logic, it becomes difficult to develop and practice professional competency. Staff are turned into technical experts without professional normative bindings, in accordance with the principles of the market and bureaucracy.^28^ As the IC is meant to be holistic,^14^ balancing relational and practical care,^46^ working with patients in settings over time implies a large proportion of emotional work. Hasenfeld argues that it is the institution that defines acceptable emotions.^29^ If the emotional work is not perceived to be sufficiently recognized in the service, and the execution of technical tasks is prioritized, this might be an important reason for the challenges the IC‐staff experience.^24^ Based on our finding, health‐care professionals should be involved in the continuous work of quality improvement. In addition to the establishment of a collaboration model between IC and the districts, a patient experience measurement (PREM) specifically designed for IC services could also be developed and implemented to evaluate the service and patient participation.

### 4.1 Methodological considerations

A strength in our study is that we interviewed the whole IC team, which provided experiences from several professional perspectives. Another strength is that we included participants from three different institutions. Our intension was to capture the different IC units’ working cultures and collaboration models within various municipal districts. In addition, the participants included in the study represented a broad range in both age, clinical experience and nationality. In line with critical realism, we can never be, nor should be, entirely free from our preconceptions. The pre‐understanding of the authors was that patient participation within geriatric health care is insufficient. During the development of interview guides and data collection process, the first author wrote memos. The fact that interviews were long and took place in the workplace makes them subject to social desirability bias. However, all interviews were held in a separate conference room located outside the ward, and within each interview, there has been an emphasis on asking about experiences to embrace both negative and positive aspects of patient participation. Further, all steps in the analysis were discussed within the author team, framed by established theory, to ensure an ongoing reflexivity and credibility. The process of analysis is attempted to be transparent and presented with clarity.^56^ The study uses a well‐known analytical strategy and has a specific study aim to ensure the information power.^67^ As the present study is part of a larger project, interviews with patients and their relatives in IC have been conducted and presented in another article. However, comparing the three perspectives might have provided a larger picture in order to understand the components of patient participation. Further, the findings from the three urban institutions might not be transferable to IC services in rural districts or in other countries. However, despite diversity in these services and different organizational models,^12^ they are comparable due to the same mechanisms with regard to purpose, structure, function and content.^68^ We thus believe our findings will have great relevance in similar IC units.

## 5 CONCLUSION

Underlying, yet powerful, mechanisms identified are the NPM‐inspired process of corporatization, the policy of deinstitutionalization and the valuing of professionalism, representing the market, bureaucracy and the profession. IC appears as an important service in the patient pathway for older people with a great potential for patient participation. Due to the influence of NPM, staff may experience constraints that prohibit them from using discretion, perceived as a threat to patient participation. Further, they may adopt routines that simplify their interactions with patients. In order to facilitate patient participation, the districts should be required to actively engage in the patient pathway through face‐to‐face meetings with the patients and relatives, and to work in teams with the health‐care professionals in IC, valuing their observations and opinions. A patient experience measurement designed for IC could be developed and implemented to evaluate the service and patient participation as part of the continuous quality improvement work. Our results call for more emphasis on diversity in an individualized rehabilitation process and an acknowledgement that psychological and social aspects are critical for patient participation in health care for older people in IC.

## CONFLICTING INTERESTS

This research received no specific grant from any funding agency in the public, commercial or not‐for‐profit sectors.

## ETHICAL CONSIDERATIONS

The study was pre‐approved and registered by the Norwegian Centre for Research Data (No. 53013). After receiving written and oral information, all the participants gave their informed consent. This included the assurance that they could withdraw their consent without consequences at any time. The audiotapes were secured in Services for Sensitive Data, an environment in compliance with Norway's Privacy and Electronic Communication Directive.
